# Acute symptomatic peri-lead edema 33 hours after deep brain stimulation surgery: a case report

**DOI:** 10.1186/s13256-017-1275-6

**Published:** 2017-04-14

**Authors:** Nathan B. Schoen, Walter J. Jermakowicz, Corneliu C. Luca, Jonathan R. Jagid

**Affiliations:** 1 Department of Neurological Surgery, University of Miami Miller School of Medicine, 1150 NW 14th St., Miami, Florida 33136 USA; 2 University of Miami Miller School of Medicine, 1150 NW 14th St., Miami, Florida 33136 USA

**Keywords:** Parkinson’s disease, Surgical complication, Steroids, Case report

## Abstract

**Background:**

Symptomatic peri-lead edema is a rare complication of deep brain stimulation that has been reported to develop 4 to 120 days postoperatively.

**Case presentation:**

Here we report the case of a 63-year-old Hispanic man with an 8-year history of Parkinson’s disease who underwent bilateral placement of subthalamic nucleus deep brain stimulation leads and presented with acute, symptomatic, unilateral, peri-lead edema just 33 hours after surgery.

**Conclusions:**

We document a thorough radiographic time course showing the evolution of these peri-lead changes and their regression with steroid therapy, and discuss the therapeutic implications of these findings. We propose that the unilateral peri-lead edema after bilateral deep brain stimulation is the result of severe microtrauma with blood–brain barrier disruption. Knowledge of such early manifestation of peri-lead edema after deep brain stimulation is critical for ruling out stroke and infection and preventing unnecessary diagnostic testing or hardware removal in this rare patient population.

## Background

Parkinson’s disease (PD) is one of the most debilitating chronic neurologic disorders and is associated with a two-fold increased risk of death from any cause [1]. Deep brain stimulation (DBS) is the surgical treatment of choice for PD and numerous studies have shown it to be a significantly more effective treatment option for moderate and advanced PD than the best medical management [1–4]. Complications after DBS are rare and typically include infections, intracranial hemorrhages, cognitive deficits, and postoperative seizures. A recently described complication of DBS is symptomatic peri-lead edema, the transient appearance of edema around a newly implanted DBS lead, typically associated with headache and mild neurological deficits. Since the first reports of this condition in 2011 over 40 cases have now been described [5–8]. These studies consistently describe peri-lead edema as appearing 4 to 120 days after surgery, being self-limiting and responsive to steroids. Clinically, peri-lead edema is important to recognize and distinguish from ischemic stroke or infection, diagnoses that may prompt further interventions or hardware removal.

In this report we are the first to present a case of symptomatic peri-lead edema appearing 33 hours after DBS surgery, nearly 3 days earlier than previously published in the literature. The patient’s early presentation allowed us to obtain a thorough radiographic analysis of the evolution of these peri-lead changes.

## Case presentation

A 63-year-old Hispanic man with a past medical history of colon cancer in remission and hypertension underwent placement of bilateral subthalamic nucleus (STN) electrodes at our institution. He had an 8-year history of idiopathic PD prior to his DBS surgery, with bradykinesia as the predominant symptom. He developed motor complications after levodopa treatment including wearing off, “delayed on,” and dyskinesias. Preoperative neurological testing with the Unified Parkinson’s Disease Rating Scale (UPDRS) showed a 62% improvement after levodopa challenge. A neuropsychological evaluation revealed no cognitive deficits. His case was evaluated by the interdisciplinary movement disorders committee at the University of Miami and he was considered a good candidate for DBS.

**Fig. 1:**
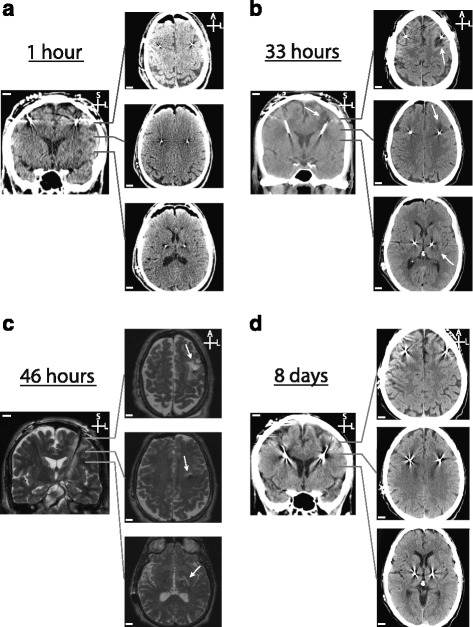
Radiographic time course of peri-lead edema. **a** Coronal and axial slices of computed tomography obtained 1 hour after surgery with no evidence of edema. **b** Computed tomography obtained 33 hours after surgery demonstrating evolving left peri-lead edema, especially along the proximal third of the deep brain stimulation lead. **c** T2-weighted MRI sequences obtained 46 hours after surgery showing significant peri-lead edema extending the entire length of the left lead. **d** Computed tomography obtained 8 days after surgery showing normal postoperative changes with no evidence of peri-lead edema. The *white arrows* highlight the peri-lead edema. Scale bar – 1 cm. *A* anterior, *L* lateral, *M* medial, *S* superior

He had bilateral STN leads placed in a single procedure. He was awake during this surgery with intravenously administered dexmedetomidine used for anesthesia. Trajectories were planned from a preoperative high resolution contrasted magnetic resonance imaging (MRI) obtained the week prior to surgery using the Medtronic StealthStation 7 System. Leads (Medtronic Model 3389, Minneapolis, MN, USA) were placed using the Integra (Plainsboro, NJ, USA) Cosman-Roberts-Wells (CRW) stereotactic frame. Microelectrode recordings and macrostimulation through the leads were used to optimize final electrode location. On both sides our patient showed significant improvement of motor symptoms with no side effects, as assessed by a movement disorders neurologist, and the first and only pass was used for final electrode placement on both sides. He received prophylactic antibiotics (vancomycin and cefepime) before and after the procedure. Postoperative computed tomography (CT) 2 hours after surgery demonstrated properly placed electrodes in the STN with no evidence of hemorrhagic or ischemic stroke or edema (Fig. 1a). He was admitted to a ward and discharged at his neurological baseline the morning after surgery with the intention of returning 1 week later for placement of DBS extension cables and generator.

Several hours after returning home, still on the first postoperative day, he developed severe headache and nausea and presented to our emergency room that evening. His headache was reported as having rapid onset, 10/10 strength, bilateral, and emanating from the top of his head. He remained neurologically intact and there was no evidence of fever. A laboratory work-up, including complete blood count (CBC), basic metabolic panel (BMP), erythrocyte sedimentation rate (ESR), C-reactive protein (CRP), and blood cultures was non-remarkable. In addition, the surgical incision was intact and showed no evidence of erythema or discharge. A CT obtained in the emergency room showed well-placed DBS leads, but with peri-lead hypodensity suggestive of edema on the left side, extending from cortex to the subcortical nuclei (Fig. 1b). The region of edema was on average 2.1 cm in diameter at the cortex and 1.5 cm in diameter when subcortical, but was not observed at the tip of the electrode. Hemorrhage or significant mass effect was not observed.

To further evaluate these findings, we next obtained a contrasted MRI of his brain (Fig. 1c). T2 sequences suggested peri-lead edema extending from the cortical surface the distance of the lead and beyond the tip, to the pars compacta. The edema was most prominent at the cortex. This dominant component of edema, along the proximal third of the electrode, was 3.6 cm in length in the anteroposterior dimension and 2.1 cm wide. The edema surrounding the distal two thirds of the electrode was cylindrical with a 1.5 cm diameter and involving the posterior limb of the internal capsule. Contrast enhancement or diffusion restriction was not observed.

Given the clinical findings, imaging, and laboratory results we ruled out ischemia or infection and presumed this to be a reactive self-limited process. He was admitted for observation and intravenously administered dexamethasone was started (10 mg bolus, followed by 4 mg every 6 hours). Ondansetron was given for nausea. By the following morning his symptoms had resolved and he was discharged home on a 6-day oral steroid taper.

At an interval follow-up 1 week later, he remained at his neurological baseline with no recent headaches or nausea. A new CT showed complete resolution of edema (Fig. 1d). He noted a subjective bilateral improvement of motor symptoms, suggestive of microlesion effect, through the entire week. He underwent the second stage generator and extension cable implants with no complications and has had good control of his PD symptoms since.

## Discussion

This case is unusual because the patient presented with peri-lead edema just 33 hours after DBS surgery. Previous literature has consistently described the edema no sooner than 4 days postoperatively [5–9]. Further, our radiographic documentation thoroughly illustrates the time course of edema development and resolution. Aside from nausea and severe headache radiating to the top of our patient’s head, which is the most typical presenting symptom in these patients, he was non-focal neurologically through the duration of his symptoms [5–8].

Since the first descriptions of peri-lead edema after DBS, this postoperative complication has perplexed the medical community. Even in cases where implants are placed bilaterally, symptoms are usually unilateral. Onset of symptoms or of radiographic edema has been reported at 4 to 120 days postoperatively [5–9]. Peri-lead edema has been identified incidentally in asymptomatic patients, but common symptoms include headache, new neurological deficit, seizures, or worsening of pre-existing symptoms. While the edema may surround the entire lead or spare the tip, seizures and worsening of neurological symptoms appear to be more common when there is a subcortical component to the edema [7]. Stroke and infection are typically ruled out first. It is unknown to what degree peri-lead edema responds to steroids, which are typically given in symptomatic cases. Most patients ultimately have complete resolution of symptoms; however, cases with persistent symptoms have been noted [5–8]. In addition, recent reports have linked peri-lead edema to the formation of cystic cavitations and both have been suggested to occur as a result of a common pathological process [9].

The most commonly proposed mechanisms for peri-lead edema have been immune hypersensitivity to lead components or microtrauma that allows cerebrospinal fluid (CSF) to track along the lead. An immunological process is inconsistent with our patient’s unilateral presentation and no evidence of allergic reaction or hypersensitivity to lead components has been identified. We believe the appearance of peri-lead edema so soon after DBS may better support mechanical trauma with blood–brain barrier disruption as the cause of this complication, since such effects would be expected soon after surgery radiographically, rather than with a delay. The unilateral nature of the edema may imply a greater degree of microtrauma severity.

## Conclusions

The clinical relevance of this report lies in the need for the medical community, whether in the emergency room or movement disorders clinic, to recognize this pathology and distinguish it from ischemic stroke or postoperative infection, which require further diagnostic testing or surgery. Previous surgical interventions in symptomatic patients with peri-lead edema and cysts have included lumbar puncture, fluid tap from the surgical site, cyst aspiration, and lead removal, typically yielding no evidence of infection [4, 10]. In addition to preventing unnecessary interventions, increased awareness of this pathology will allow us to improve our study of the etiology and relevance of this rare complication.

## Abbreviations

BMP: Basic metabolic panel
CBC: Complete blood count
CRP: C-reactive protein
CSF: Cerebrospinal fluid
CT: Computed tomography
DBS: Deep brain stimulation
ESR: Erythrocyte sedimentation rate
PD: Parkinson’s disease
STN: Subthalamic nucleus
UPDRS: Unified Parkinson’s Disease Rating Scale
